# Spontaneous mutations and mutational responses to penicillin treatment in the bacterial pathogen *Streptococcus pneumoniae* D39

**DOI:** 10.1007/s42995-024-00220-6

**Published:** 2024-04-16

**Authors:** Wanyue Jiang, Tongtong Lin, Jiao Pan, Caitlyn E. Rivera, Clayton Tincher, Yaohai Wang, Yu Zhang, Xiang Gao, Yan Wang, Ho-Ching T. Tsui, Malcolm E. Winkler, Michael Lynch, Hongan Long

**Affiliations:** 1 Institute of Evolution and Marine Biodiversity, KLMME, Ocean University of China, Qingdao, 266003 China; 2 Laboratory for Marine Biology and Biotechnology, Laoshan Laboratory, Qingdao, 266237 China; 3 Department of Biology, Indiana University, Bloomington, IN 47405 USA; 4 School of Mathematics Science, Ocean University of China, Qingdao, 266000 China; 5 State Key Laboratory of Microbial Technology, Microbial Technology Institute, School of Life Science, Shandong University, Qingdao, 266237 China; 6 Biodesign Center for Mechanisms of Evolution, Arizona State University, Tempe, AZ 85281 USA

**Keywords:** Neutral evolution, Mutation spectrum, Antibiotics, DNA mismatch repair, Oxidative damage repair

## Abstract

Bacteria with functional DNA repair systems are expected to have low mutation rates due to strong natural selection for genomic stability. However, our study of the wild-type *Streptococcus pneumoniae* D39, a pathogen responsible for many common diseases, revealed a high spontaneous mutation rate of 0.02 per genome per cell division in mutation-accumulation (MA) lines. This rate is orders of magnitude higher than that of other non-mutator bacteria and is characterized by a high mutation bias in the A/T direction. The high mutation rate may have resulted from a reduction in the overall efficiency of selection, conferred by the tiny effective population size in nature. In line with this, *S. pneumoniae* D39 also exhibited the lowest DNA mismatch-repair (MMR) efficiency among bacteria. Treatment with the antibiotic penicillin did not elevate the mutation rate, as penicillin did not induce DNA damage and *S. pneumoniae* lacks a stress response pathway. Our findings suggested that the MA results are applicable to within-host scenarios and provide insights into pathogen evolution.

**Supplementary Information:**

The online version contains supplementary material available at 10.1007/s42995-024-00220-6.

## Introduction

*Streptococcus pneumoniae* is a human nasopharyngeal pathogen that causes diseases, including strep throat, pneumonia, meningitis, and otitis media, particularly in young children and the elderly. The virulence factors and pathogenesis of this Gram-positive pathogenic bacteria have been extensively studied (Kadioglu et al. 2008; Mitchell and Mitchell 2010; Musher 1992; Van der Poll and Opal 2009). However, information on how the initial nasopharyngeal colonization process influences within-host evolution and future transmission, is currently lacking (Chaguza et al. 2020; Green et al. 2021). Within the hosts, the evolution of this species is driven by multiple selective forces (clonal expansion, transmission pressure, host immunity), which may complicate the evolutionary patterns of this pathogen. To reveal the evolutionary patterns of *S. pneumoniae*, we examined spontaneous mutational features at the whole-genome level without strong selective drivers. To do so, we conducted multiple mutation accumulation experiments (MA) combined with whole-genome-sequencing (WGS) (Keightley and Halligan 2009; Lee et al. 2012; Lynch et al. 2008).

Cells possess multiple mechanisms to repair DNA damage and thus prevent mutations. However, the repair patterns of canonical repair pathways remain unclear in *S. pneumoniae*. One such pathway is DNA mismatch repair (MMR), which is composed of a few well-conserved proteins (MutS/MutL/MutH), functioning in the post-replicative repair of mismatched DNA (Jun et al. 2006; Kolodner and Marsischky 1999; Kunkel and Erie 2005; Li 2008; Modrich and Lahue 1996). Other repair pathways, involved with a nucleotide triphosphate hydrolase MutT (named as MutX in *S. pneumoniae*) and an A/G-specific adenine glycosylase MutY play important roles in repairing DNA oxidative damages (Ames and Gold 1991; Mejean et al. 1994; Nghiem et al. 1988; Tajiri et al. 1995). Considering that *S. pneumoniae* is catalase-deficient (Pericone et al. 2002), it might be subjected to high mutation pressure. Uncovering the repair patterns of the MMR and the oxidative damage repair pathways (MutX and MutY) can provide insights into the mutation spectrum determinants and the long-term evolution of *S. pneumoniae*.

During clinical treatments of diseases caused by *S. pneumoniae* an increasing number of strains have developed resistance to clinically-recommended antibiotics such as the β-lactam penicillin, one of the most widely-used antibiotics for treating *S. pneumoniae* (Jacobs 1992; Van de Beek et al. 2006; Van der Poll and Opal 2009; Yu et al. 2003). Currently, our knowledge of the mutational processes underlying acquisition of resistance under penicillin exposure is limited for this pathogen. Particularly lacking is information on the influence of penicillin on the mutation rate, as opposed to the ability of natural selection to promote resistance mutations. It has been reported that bacteria are often exposed to sublethal (i.e., subinhibitory) concentrations of certain antibiotics, which can not only promote the emergence and spread of resistant bacteria in humans and animals but may even induce multi-drug resistant mutations, posing a threat to human health (Andersson and Hughes 2014; Baquero et al. 2008; Carmeli et al. 1999; Jiang et al. 2013; Jørgensen et al. 2013; Long et al. 2016; Zurek and Ghosh 2014). De novo mutations are the main source of antibiotic resistance (Woodford and Ellington 2007). Based on above, uncovering the effects of penicillin treatment on genome-wide mutations of *S. pneumoniae* may provide essential guidance for clinical disease treatment.

In this study, we determined sublethal concentrations of penicillin in vitro on the wild-type *S. pneumoniae* D39 and three mutant strains constructed from the wild-type: Δ*mutS* (MMR-deficient), Δ*mutX* (MutX-deficient) and Δ*mutY* (MutY-deficient). Then, we applied the MA-WGS strategy to investigate spontaneous mutational features at whole-genome level and the repair patterns of the classical DNA damage repair systems, as well as the effects on genomic mutations upon penicillin treatment. Our results uncovered the spontaneous mutational features of *S. pneumoniae* in different genetic backgrounds at the whole-genome level, including the rate, molecular spectrum of genomic mutations, and repair patterns of the aforementioned repair systems. This study also provided quantitative insights into the response of this pathogen to penicillin treatment.

## Materials and methods

### Bacterial strains and growth conditions

*Streptococcus pneumoniae* strains were derived from the encapsulated serotype-2 D39W progenitor strain IU1690 (Lanie et al. 2007; Slager et al. 2018). For cultures in liquid broth, bacteria were grown statically in Becton-Dickinson brain heart infusion (BHI) broth at 37 °C in an atmosphere of 5% CO_2_, and growth was monitored by OD620 as described before (Tsui et al. 2016). During growth for mutation accumulation (MA) procedure, colonies were grown at 37 °C, 5% CO_2_ on brain–heart infusion agar plates (BHI, Oxoid) supplemented with 5% (vol/vol) defibrinated sheep blood. Streaked colonies on BHI agar plates appeared as ~ 1 mm-diameter smooth colonies with a grass–green colored hemolytic ring around them. Stock solutions of penicillin G sodium salt (Cat. No.: P3032-10MU, Sigma–Aldrich) were made according to the manufacturers’ instructions. We followed biosafety level 2 procedures as stated in the bioprotocol 15-038 (Institutional Biosafety Committee, Indiana University at Bloomington). Details on construction of deletion mutant strains are in Supplementary Table S1, following previously published procedures (Ramos-Montanez et al. 2008; Tsui et al. 2011, 2014, 2016).

### Determining sublethal concentrations of penicillin for *S. pneumoniae* D39 in vitro

To determine the range of sublethal concentrations of penicillin used for further experiments, we generated survival curves by measuring the efficiency of plating (EOP). These curves were established as follows. Cells were inoculated from frozen glycerol stocks into BHI broth, serially diluted, and incubated 12–15 h statically at 37 °C in an atmosphere of 5% CO_2_. The next day, cultures at OD620 ≈ 0.1–0.4 were diluted to OD620 ≈ 0.005 in BHI broth. They were then plated onto BHI agar plates with gradients of penicillin concentrations: 0, 0.002, 0.004, 0.006, 0.008, 0.01, 0.012, 0.014, 0.016 ng/μL (each group was with four replicates). Plates were maintained at 37 °C and 5% CO_2_ for 24 h, after which colony forming units (CFU) were counted. The EOP was then calculated as *m*/*N*, where *m* is the CFU of the plate containing 0–0.016 ng/μL penicillin, and *N* is the CFU of the penicillin-free plate.

### Mutation accumulation (MA) procedures

To explore the whole-genome mutational features and mutational responses to penicillin treatment, we then performed MA experiments on the four strains (wild-type, Δ*mutS*, Δ*mutX*, and Δ*mutY*). For each strain, to initiate the MA lines, cells from an ancestral colony were plated onto a BHI plate without penicillin. The grown colonies were then streaked onto BHI plates containing 0, 0.002, 0.004, 0.006, and 0.008 ng/μL. Number of MA lines of different strains for each penicillin concentration was not balanced: for the wild-type *n* = 24; for Δ*mutS* *n* = 8; for Δ*mutX* and Δ*mutY* *n* = 16. All MA lines were transferred every 24 h by streaking single colonies.

The MA experiments lasted 45–52 days. The number of cell divisions between transfers was estimated every ~ 3 weeks by cutting single colonies of five randomly-picked lines from agar plates, serially diluted in 1 × PBS to a suitable cell density, plated on penicillin-free plates, and then CFU was determined as outlined above (see Supplementary Table S1). The effective population size (*N*_e_) during MA experiments of each treatment was calculated by taking the harmonic mean of successive doublings from a population size of 1 until the final population size was reached (Wahl and Gerrish 2001).

### DNA extraction, library construction, and sequencing

The genomic DNA of the wild-type ancestor strain and all the 320 MA lines were extracted with the Wizard Genomic DNA Purification Kit (Promega). A Nextera DNA Library Preparation Kit (Illumina) was used to construct the genomic DNA libraries with an insert size of 500 bp for Illumina Hiseq 2500 PE250 sequencing; this led to 128 × (SE: 4.13) mean depth of sequencing coverage and 98.85% (SE: 0.23%) of the genome covered with high-quality reads, using the chromosome sequence of the model strain *S. pneumoniae* D39 (NC_008533.2) as the reference (Table 1; Supplementary Table S2). 18 MA lines with coverage of less than 20 × or cross-contamination were removed (Supplementary Table S3).

**Table 1:** MA experimental details

Strain	Penicillin (ng/μL)	*N*	Transfers	Division	*N*_e_	Depth (×)
Wild-type	0 (WT_A)	23	51	884	9.00	106
Wild-type	0.002 (WT_B)	24	52	884	9.00	110
Wild-type	0.004 (WT_C)	22	52	854	8.50	98
Wild-type	0.006 (WT_D)	22	52	845	8.50	142
Wild-type	0.008 (WT_E)	24	50	829	9.00	133
Δ*mutS*	0 (mutSA)	7	50	879	9.50	103
Δ*mutS*	0.002 (mutSB)	7	52	897	9.00	109
Δ*mutS*	0.004 (mutSC)	8	52	857	8.50	146
Δ*mutS*	0.006 (mutSD)	8	52	863	9.00	118
Δ*mutS*	0.008 (mutSE)	8	50	840	9.00	105
Δ*mutX*	0 (mutXA)	16	47	788	9.00	131
Δ*mutX*	0.002 (mutXB)	15	47	772	9.00	150
Δ*mutX*	0.004 (mutXC)	15	48	791	9.00	149
Δ*mutX*	0.006 (mutXD)	15	47	790	9.00	124
Δ*mutX*	0.008 (mutXE)	16	45	732	8.50	152
Δ*mutY*	0 (mutYA)	13	50	840	9.00	129
Δ*mutY*	0.002 (mutYB)	15	49	756	8.00	130
Δ*mutY*	0.004 (mutYC)	15	49	811	8.50	163
Δ*mutY*	0.006 (mutYD)	14	48	817	9.00	142
Δ*mutY*	0.008 (mutYE)	15	48	742	8.50	122

*N*, number of replicate MA lines; Transfers, mean number of transfers during the experiment; Division, mean cell divisions in the final analyses; *N*_*e*_, effective population size of each penicillin-treatment corresponded to a certain concentration; Depth (×), depth of sequencing coverage

### Mutation analyses

After trimming adapters of the raw data by Trimmomatic (v-0.36) (Bolger et al. 2014), the trimmed reads of each MA line were mapped to the reference genome (NCBI Genome accession No.: NC_008533.2) with Burrows-Wheeler Aligner (v-0.7.17) mem (Li and Durbin 2009). The HaplotypeCaller module in Genome Analysis Toolkit (GATK v-4.1.2) was used for calling base-pair substitutions (BPSs) and short indels using GATK's best practices recommendations, and only unique BPSs were considered (DePristo et al. 2011; McKenna et al. 2010). The short sequence reads of MA lines of each group were mapped to the D39 reference genome to identify structural variations in Breseq (v-0.35.1) (Deatherage and Barrick 2014). Validation of BPSs and indels with Integrative Genomics Viewer (IGV v-2.8.2) was also performed (Thorvaldsdóttir et al. 2013). Genome-wide BPS mutation rate μ (per nucleotide site per cell division) was calculated by the following formula:

$$\frac{s}{{\mathop \sum \nolimits_{1}^{n} N \times T}},$$

where *s* is the total number of mutations across all MA lines, *n* is the total number of MA lines, *N* is the analyzed sites in one MA line, and *T* is the number of cell divisions passed in the entire mutation accumulation process of one MA line. BPS mutation rate of each gene was also calculated using the above formula, in which *s* is the total number of mutations carried by a certain gene, *n* is the total number of MA lines, *N* is the base number of the gene, and *T* is the number of cell divisions passed in the entire mutation accumulation process of one MA line.

The repair efficiency was calculated by the following formula:

$$\frac{{\mu_{{{\text{mutant}}}} - \mu_{{\text{wildtype }}} }}{{\mu_{{{\text{mutant}}}} }},$$

where *μ*_mutant_ was the BPS mutation rate of Δ*mutS*, Δ*mutX*, or Δ*mutY* and *μ*_wildtype_ was the BPS mutation rate of the wild-type.

The mutation bias *m* was calculated by ${m=\mu }_{G:C\to A:T+G:C\to T:A}/{\mu }_{A:T\to G:C+A:T\to C:G}$, and the transition to transversion ratios (ts/tv; *n* is the total number of MA lines) with the following formula:

$$\frac{{\mathop \sum \nolimits_{1}^{n} {\text{transitions}}}}{{\mathop \sum \nolimits_{1}^{n} {\text{transversions}}}}.$$

### Calculation of population genetic parameters

To estimate the effective population size (*N*_e_), we downloaded 196 natural strains of *S. pneumoniae* with different serotypes from the NCBI SRA database (Supplementary Table S4). We used Unicycler (v-0.4.8) (Wick et al. 2017) to assemble the draft genomes of all the natural strains, then blasted their crucial genes related to multiple DNA repair pathways—*mutS*, *mutL*, *mutM*, *mutX*, and *mutY* with the gene sequences of *S. pneumoniae* D39. One strain was then removed due to incomplete crucial genes, as we required all strains to have intact reading frames of these genes, and a total of 195 strains met the conditions. Then we used BWA (v-0.7.12) (Li and Durbin 2009) to align the raw reads of the 195 natural strains to the chromosome of *S. pneumoniae* D39 (NCBI Genome accession No.: NC_008533.2) and calculated *π*_s_, the average pairwise genetic distance at four-fold degenerate sites using the following formula:

$$\pi_{{\text{s}}} = \frac{n}{n - 1} \times (1 - \Sigma \pi^{2} ),$$

where *π* is the average number of nucleotide differences per site between two DNA sequences in all possible pairs of the strains, *n* is the number of strains. *N*_e_ of *S. pneumoniae* was calculated using the following formula, where *μ* is BPS mutation rate per nucleotide site per cell division:

$$N_{{\text{e}}} \approx \frac{{\pi_{{\text{s}}} }}{2\mu }.$$

### Gene expression analysis based on RNAseq

RNAseq data of *S. pneumoniae* D39 wild-type strain were retrieved from NCBI SRA with the BioProject Number of PRJNA6952 (SRR13563519–SRR13563522) (Hirschmann et al. 2021). Raw reads of the four parallel samples were trimmed with default parameters of Fastp (v-0.20.1) (Chen et al. 2018), and an index of the genome from the file NC_008533.2 (*S. pneumoniae* D39) was created with Hisat2 (v-2.2.1) (Kim et al. 2015). Gene expression levels in these samples were analyzed with StringTie (v-2.1.5) (Pertea et al. 2016).

## Results

### Neutrality tests on the mutation accumulation datasets

**Fig. 1:**
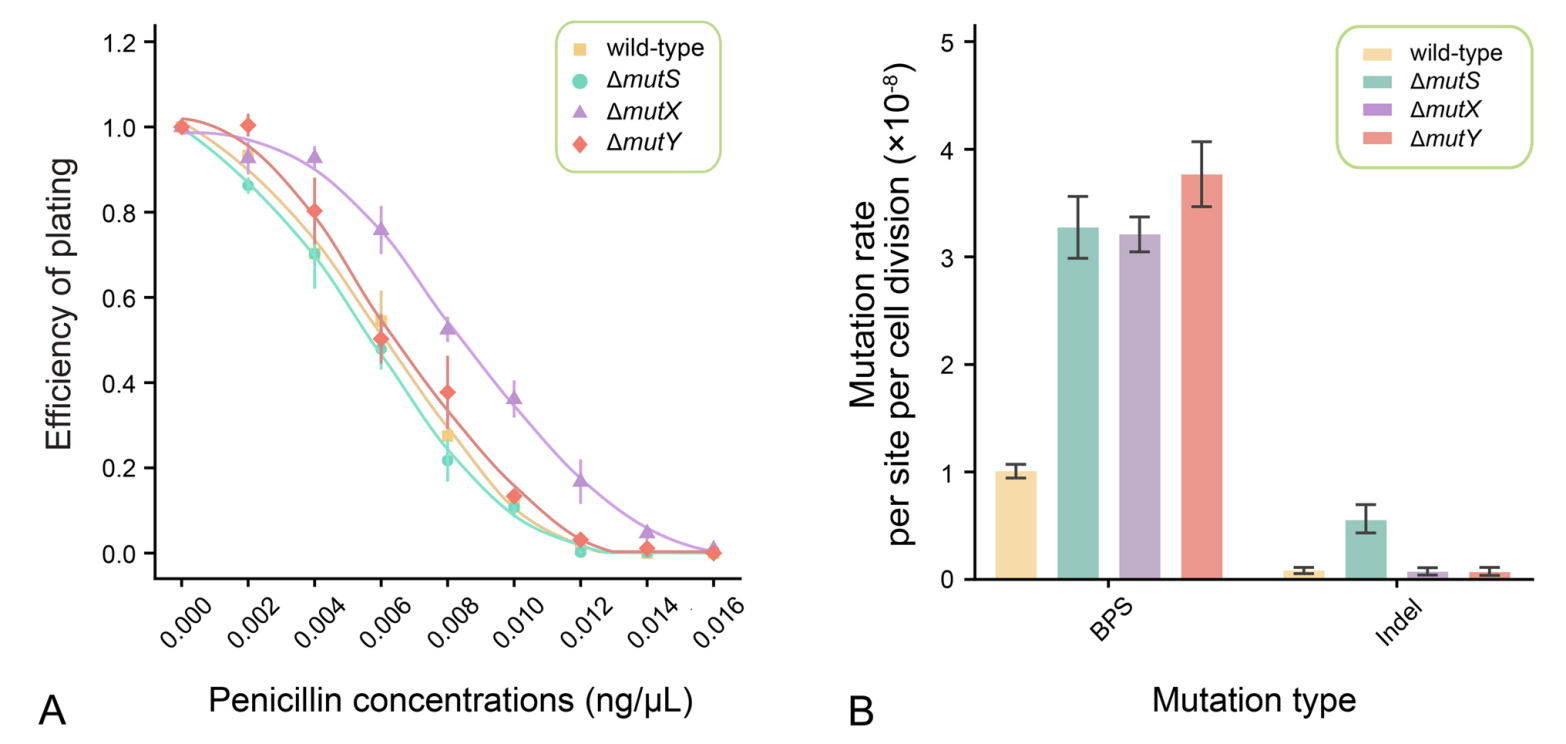
Efficiency of plating (EOP) and mutation rates. **A** Efficiency of plating (EOP) of the four strains under nine penicillin concentrations (points are means, error bars denote SE; *n* = 4). **B** BPS (base-pair-substitution) and indel (insertion and deletion) mutation rates of the penicillin-free MA lines of the four strains (error bars denote 95% Poisson confidence intervals)

Survival curves indicated that penicillin concentrations below 0.008 ng/μL had sublethal effects (Fig. 1A), and mutation accumulation experiments (MA) were performed at this level and below (Table 1; Supplementary Tables S5, S6). We compared the nonsynonymous/synonymous mutation ratio of MA lines of each strain with the random expectation and found no evidence of mutations being biased by selection in the penicillin-free MA lines (*P* > 0.05, *χ*^2^ test; Supplementary Tables S7–S10). Furthermore, the nonsynonymous/synonymous base-pair substitutions (BPSs) ratio in the control and penicillin-treated groups did not differ significantly (*χ*^2^ test, *P* > 0.05 in all cases), indicating that the majority of acquired amino acid-altering BPSs were not selectively promoted by penicillin treatment but simply accumulated in a neutral fashion.

However, we identified three genes in the wild-type control MA lines with more mutations than the random expectation (Supplementary Tables S11, S12): an aminotransferase of the DegT/DnrJ/EryC1/StrS family (gene ID: SPD_RS08630), a transcriptional regulator of the GntR family (SPD_RS00325), and a member of the glycosyltransferase family 1 (SPD_RS01740). No selection was detected in SPD_RS01740 (*P* = 0.53, exact binomial test for goodness of fit; nonsynonymous/synonymous sites ratio = 3.68, as expected, with the transitions/transversions ratio considered; Supplementary Table S7). However, all 10 and three BPSs, carried by the SPD_RS08630 and SPD_RS00325, respectively, were nonsynonymous. The high proportion of nonsynonymous BPSs in these two genes might indicate strong positive selection acting on them during the MA process (Andreani et al. 2019; Fineran et al. 2005; Hillerich and Westpheling 2006). Therefore, we excluded the BPSs of the two strongly selected genes (SPD_RS08630 and SPD_RS00325) from the mutational feature calculation, although this barely changes any pattern.

### The features of *S. pneumoniae* D39 spontaneous mutations at the whole-genome level

We detected 415 BPSs in the wild-type MA lines without penicillin treatment, resulting in a mean BPS mutation rate of 1.01 × 10^–8^ (CI: 9.13 × 10^–9^, 1.11 × 10^–8^) per nucleotide site per cell division, or ~ 0.02 per genome per cell division (Table 2; Fig. 1B; Supplementary Tables S2, S13, S14). Additionally, we found 35 indels (9 insertions and 26 deletions), with an insertion/deletion ratio of 0.35, yielding a mean small-scale indel rate of 8.50 × 10^–10^ (CI: 5.92 × 10^–10^, 1.18 × 10^–9^) per nucleotide site per cell division, which represented 8.43% of the BPS mutation rate (Table 2; Fig. 1B). The ratio of insertions/deletions indicates a deletion bias, which was consistent with most studied bacteria (Long et al. 2018a). We observed 10 indels within simple sequence repeats (SSR), which were tandem repeats of short DNA nucleotides, such as homopolymer runs and microsatellite repeats. These were usually generated by unequal crossover or polymerase slippage during replication of SSRs (Levinson and Gutman 1987).

**Table 2:** Mutation information

Strain	Penicillin (ng/μL)	BPSs	*μ*_BPS_	ts/tv	AT bias	Indels	*μ*_Indel_	Ins/Del
Wild type	0 (WT_A)	415	1.01	1.10	7.02	35	0.09	0.35
Wild type	0.002 (WT_B)	388	0.90	1.85	7.30	49	0.11	0.48
Wild type	0.004 (WT_C)	241	0.63	1.70	5.27	24	0.06	0.50
Wild type	0.006 (WT_D)	257	0.68	2.13	4.60	31	0.08	0.41
Wild type	0.008 (WT_E)	209	0.52	1.87	5.44	25	0.06	0.79
Δ*mutS*	0 (mutSA)	407	3.27	4.48	2.66	72	0.58	0.76
Δ*mutS*	0.002 (mutSB)	472	3.72	6.46	2.28	90	0.71	0.91
Δ*mutS*	0.004 (mutSC)	461	3.32	7.23	1.96	77	0.56	1.14
Δ*mutS*	0.006 (mutSD)	368	2.63	9.49	2.57	73	0.52	0.83
Δ*mutS*	0.008 (mutSE)	419	3.09	8.84	1.99	73	0.54	0.78
Δ*mutX*	0 (mutXA)	818	3.21	0.26	0.44	19	0.07	0.46
Δ*mutX*	0.002 (mutXB)	773	3.30	0.20	0.49	13	0.06	0.44
Δ*mutX*	0.004 (mutXC)	661	2.76	0.25	0.46	16	0.07	0.60
Δ*mutX*	0.006 (mutXD)	593	2.48	0.23	0.60	10	0.04	0.25
Δ*mutX*	0.008 (mutXE)	463	1.96	0.22	0.36	7	0.03	6.00
Δ*mutY*	0 (mutYA)	831	3.77	0.10	68.21	16	0.07	0.14
Δ*mutY*	0.002 (mutYB)	1056	4.61	0.12	51.50	13	0.06	0.86
Δ*mutY*	0.004 (mutYC)	1020	4.15	0.10	79.88	13	0.05	0.30
Δ*mutY*	0.006 (mutYD)	872	3.78	0.11	43.82	24	0.10	0.50
Δ*mutY*	0.008 (mutYE)	727	3.23	0.11	38.76	19	0.08	0.27

BPSs, total number of base-pair substitutions in the MA lines; *μ*_BPS_, base-pair substitution mutation rate; ts/tv, the ratio of transitions/transversions; AT bias, mutation bias in the A/T direction, (see Materials and methods for details); Indels, number of insertions and deletions; *μ*_Indel_, indel mutation rate; Ins/Del, the ratio of insertions/deletions. All mutation rates are in units of × 10^–8^ per nucleotide site per cell division

**Fig. 2:**
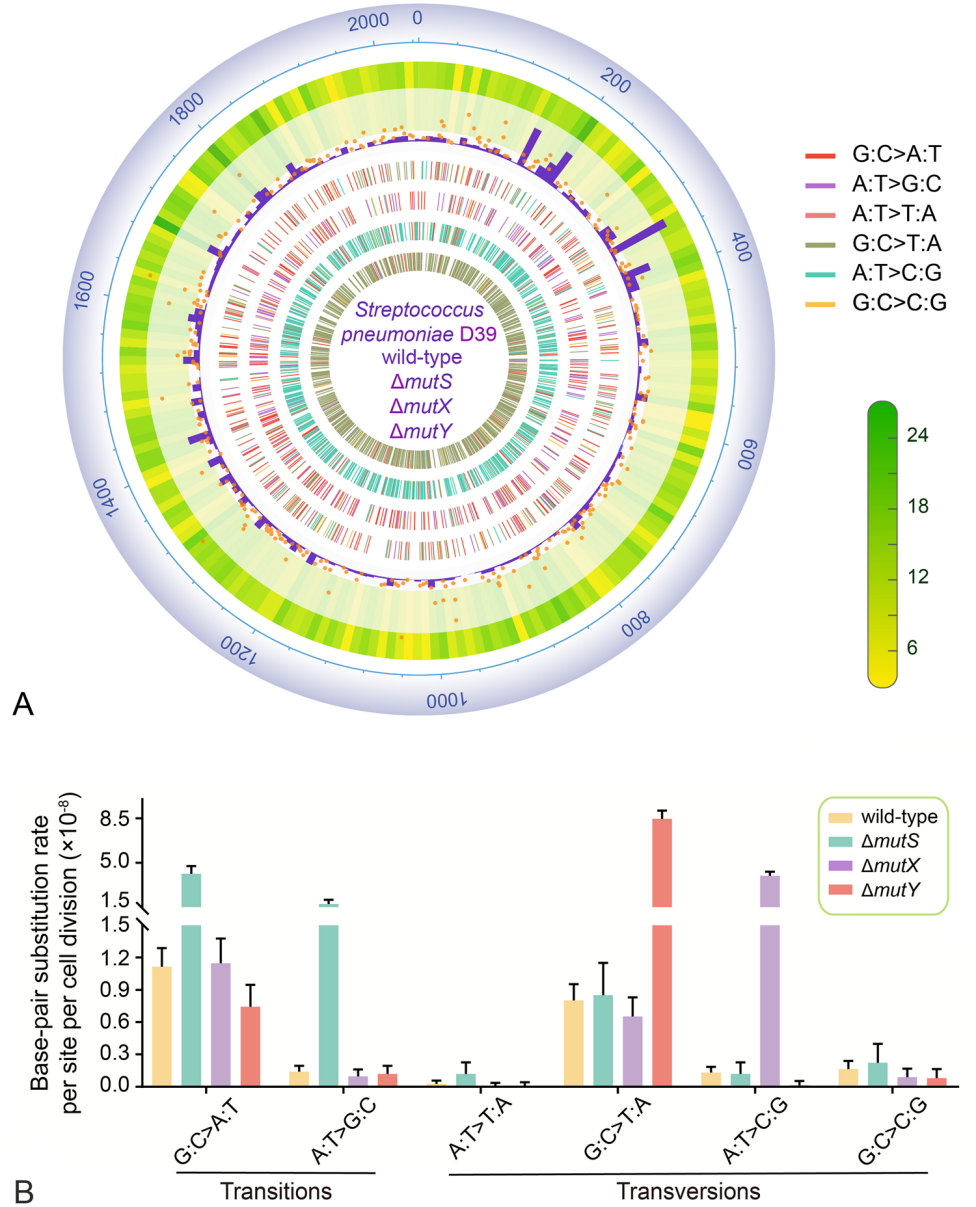
Mutational features of penicillin-free MA lines of the four strains. **A** Summary of genome-wide distribution of mutations (plotted by TBtools and R package ‘circlize’, Chen et al. 2020; Gu et al. 2014). Genome coordinates are in kbp. Circles from the outside inward: the gene density per 10-kbp bin (the whole genome is divided into 205 bins; the legend is on the left); the histogram in purple is the gene expression levels in fragments per kilobase million (FPKM) per 10-kbp bin, and the orange dots represent mutation rate per nucleotide site per cell division of each gene of the wild-type (the lowest: 6.43 × 10^–9^; the highest: 4.01 × 10^–7^); the next four inner circles with colored tiles represent the genomic location of BPSs of wild-type, Δ*mutS*, Δ*mutX* and Δ*mutY* MA lines respectively (the legend is on the right).** B** Mutation spectra of penicillin-free MA lines of the four strains. Error bars denote 95% Poisson confidence intervals

The within-line rates of different mutation types exhibited significant differences (ANOVA, *F* = 54, *P* < 0.0001). Specifically, the mutation rates of G:C→A:T transitions and G:C→T:A transversions were significantly higher than other types of mutations (Tukey test, *P* < 0.0001; Fig. 2A, B; Supplementary Table S15). The ratio of transitions/transversions (ts/tv) was 1.10, resulting in an extreme mutation bias in the A/T direction of 7.02 (mutation bias in the A/T direction was calculated as: ${m=\mu }_{G:C\to A:T+G:C\to T:A}/{\mu }_{A:T\to G:C+A:T\to C:G}$; Table 2). The mutation spectrum was characterized by a high G:C→T:A transversion rate and a relatively low A:T→G:C transition rate.

### Specificity of MMR and oxidative damage repair systems

To quantify the repair specificity of the major DNA repair systems in *S. pneumoniae*, we compared the mutational features of MMR-deficient Δ*mutS* and two oxidative-damage-repair deficient strains Δ*mutX* and Δ*mutY* with those of the wild-type (Table 1). The genome-wide BPS mutation rate of the Δ*mutS* strain, estimated from the MA lines, was 3.27 × 10^–8^ (CI: 2.96 × 10^–8^, 3.60 × 10^–8^) per nucleotide site per cell division, which was 3.24 × higher than that of the wild-type. This indicated that MMR repairs 69.17% of genome-wide pre-mutations derived from mismatched nucleotides (Table 2; Fig. 1B; Supplementary Tables S2, S13, S16). In addition, the small-scale indel mutation rate of the Δ*mutS* strain was 5.78 × 10^–9^ (CI: 4.52 × 10^–9^, 7.28 × 10^–9^) per nucleotide site per cell division, which was a 6.80 × elevation from the wild-type. The insertions/deletions ratio was 0.76 (vs. 0.35 in the wild-type). We then calculated the repair efficiency of MMR on indels, insertions, and deletions, yielding values of 85.3%, 91.2% and 80.8%, respectively (Table 2; Fig. 1B; Supplementary Tables S2, S13, S17). These results indicated a preference for MMR in repairing insertions.

The MA results showed that the genomic BPS mutation rates of the oxidative-damage-repair deficient strains Δ*mutX* and Δ*mutY* were 3.21 × 10^–8^ (CI: 2.99 × 10^–8^, 3.44 × 10^–8^) and 3.77 × 10^–8^ (CI: 3.52 × 10^–8^, 4.03 × 10^–8^) per nucleotide site per cell division respectively, which were 3.18 and 3.74 × higher than that of the wild-type. These resulted in the overall repair efficiencies of 68.6% and 73.3% by MutX and MutY, respectively (Table 2; Fig. 1B; Supplementary Tables S2, S13, S16). The small-scale indel mutation rates of the Δ*mutX* and Δ*mutY* strains were 7.45 × 10^–10^ (CI: 4.49 × 10^–10^, 1.16 × 10^–9^) and 7.26 × 10^–10^ (CI: 4.15 × 10^–10^, 1.18 × 10^–9^) per nucleotide site per cell division, which were similar to that of the wild-type, reflecting that oxidative-damage-repair systems in *S. pneumoniae* did not repair indels (Table 2; Fig. 1B; Supplementary Tables S2, S13, S17).

The mutation spectrum of the Δ*mutS* strain was typical of hypermutator bacteria with dysfunctional MMR, with significantly increased transitions, while transversions other than A:T→T:A were not (Mann Whitney test, G:C→A:T, *P* < 0.0001; A:T→G:C, *P* < 0.0001; A:T→T:A, *P* = 0.0027; Fig. 2A, B; Supplementary Table S15). 73.03% G:C→A:T and 91.98% A:T→G:C transitions were repaired by MMR, and 76.48% for A:T→T:A transversions (Supplementary Table S17). The inflated transition rates led to a transition/transversion ratio of 4.48 ×, higher than that of the wild-type, reflecting that MMR of *S. pneumoniae* preferentially repairs transitions. The mutation bias of the Δ*mutS* MA lines in the A/T direction was 2.66, which was much weaker than the 7.02 of the wild-type (Table 2).

Compared to the wild-type, A:T→C:G transversions are significantly increased in the Δ*mutX* MA lines (ANOVA, *F* = 171, *P* < 0.001; Tukey test, *P* < 0.0001), leading to ~ threefold increase in genome-wide mutation rate, a ts/tv ratio of 0.19, and mutations biased in the G/C direction (*m* = 0.44; Table 2; Fig. 2A, B). The repair efficiency of MutX for A:T→C:G transversions was 96.7% (Supplementary Table S17). In the Δ*mutY* MA lines, G:C→T:A transversions were the most significantly elevated mutation type (ANOVA, *F* = 118, *P* < 0.001; Tukey test, *P* < 0.0001), yielding a ts/tv of 0.11 and an extreme A/T mutation bias of ~ 68. The repair efficiency for G:C→T:A transversions was 90.52%, supporting that MutY specifically repairs G:C→T:A transversions (vs. 98.65% of *E. coli*; Table 2; Fig. 2A, B; Supplementary Table S17).

### Penicillin treatment caused various effects on mutational features of different strains

**Fig. 3:**
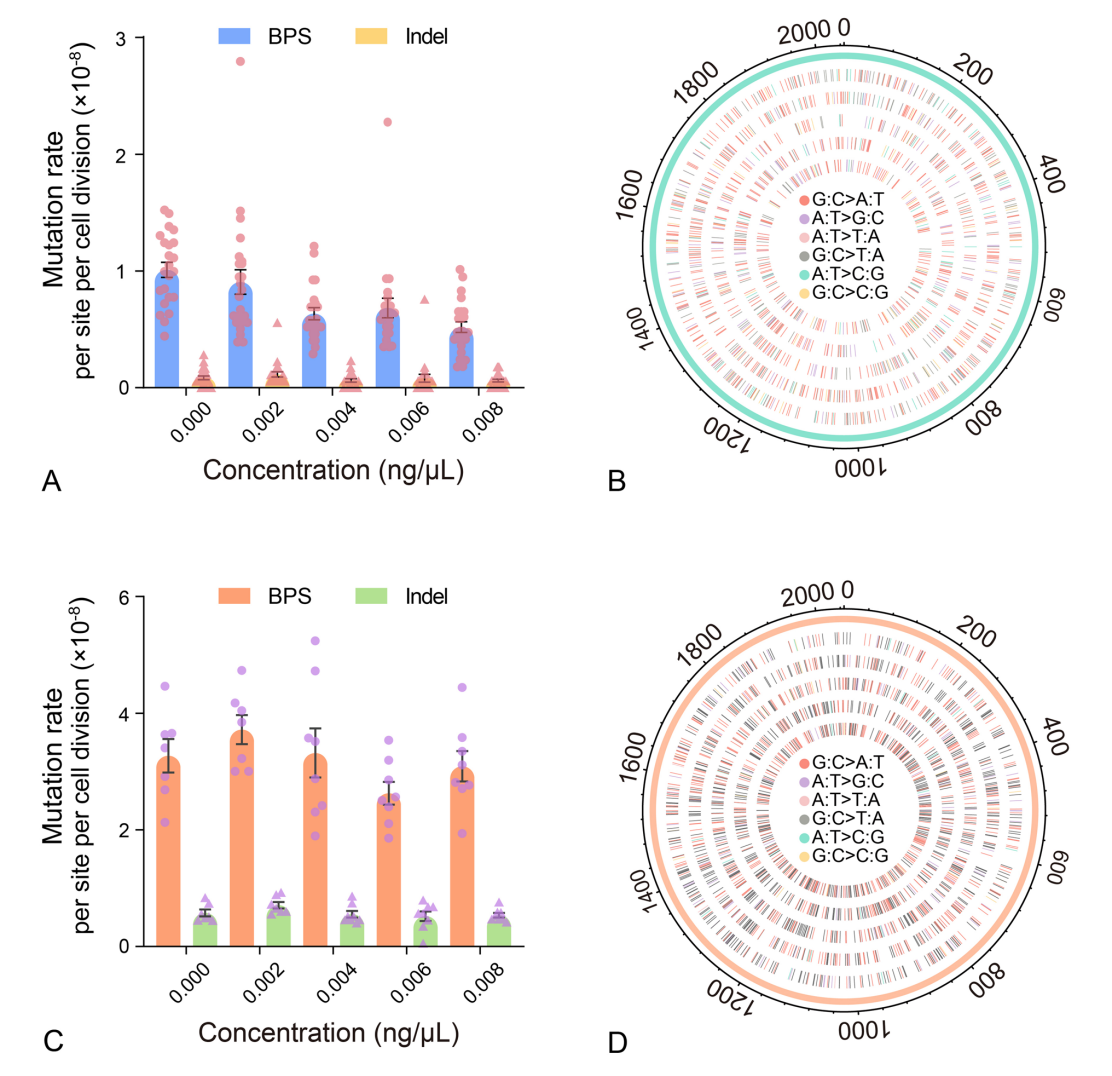
Mutational responses to penicillin treatment of the wild-type and the Δ*mutS* MA lines. **A** BPS and indel mutation rates treated with different penicillin concentrations of the wild-type MA lines. **B** The distribution of the BPS mutations on the whole genome of the wild-type MA lines. **C** BPS and indel mutation rates per nucleotide site per cell division treated with different penicillin concentrations of the Δ*mutS* MA lines. **D** The distribution of the BPS mutations on the whole genome of the Δ*mutS* MA lines. Dots and the triangles represent BPS and indel mutation rates of each MA line; error bars denote SE (**A, C**). Genome coordinates are in kbp; circles with colored tiles from outside to inside represent BPSs for 0, 0.002, 0.004, 0.006 and 0.008 ng/μL-penicillin-treated MA lines (**B, D**)

**Fig. 4:**
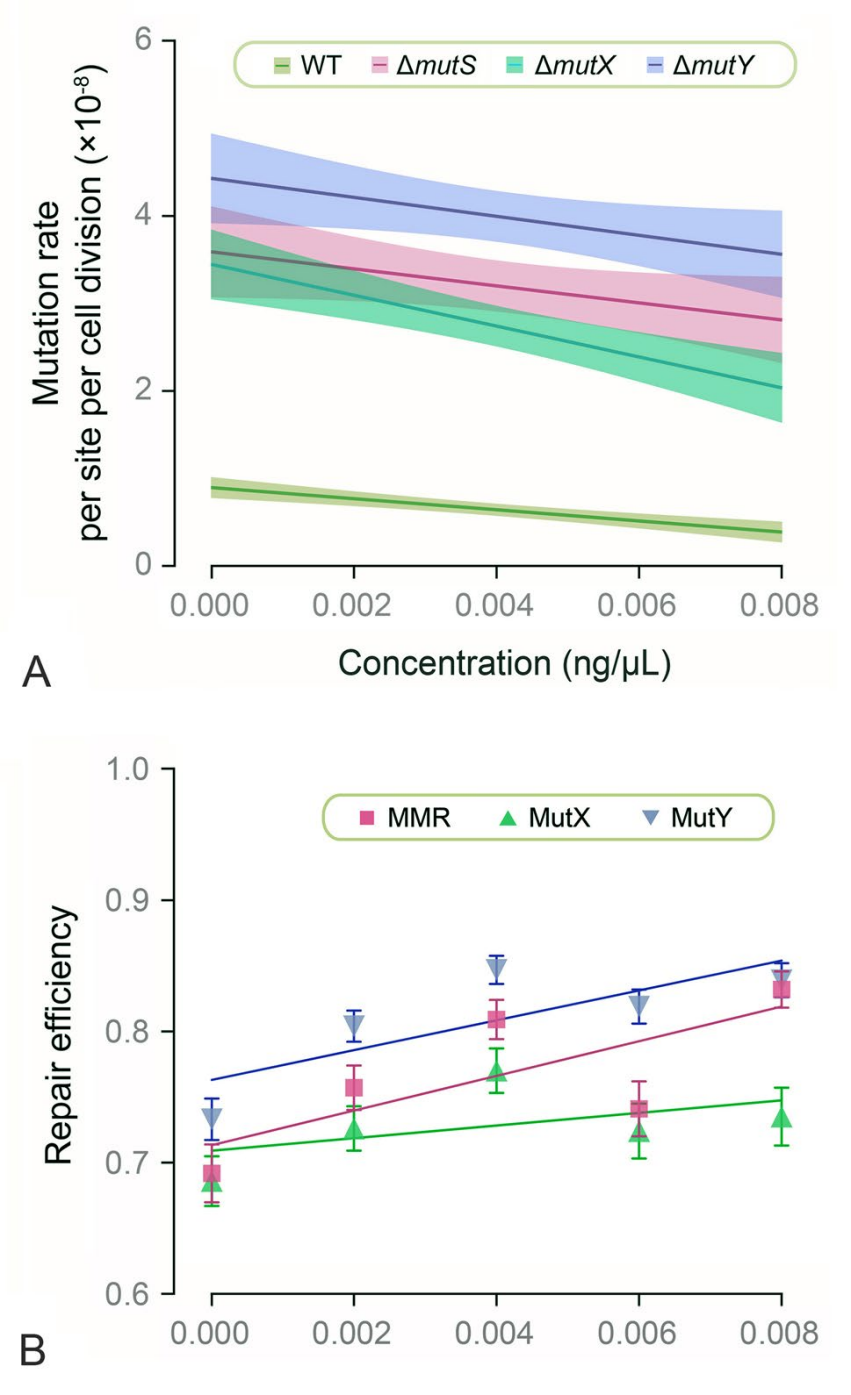
Statistical analyses of the BPS mutation rates (**A**) and repair efficiencies (**B**) in response to penicillin does. **A** Regression analysis of the BPS mutation rates against variable penicillin concentration across the four strains using linear models. Each line represents the linear regression fit for each strain, with shaded areas indicating 95% confidence intervals: *y* = (− 6.02 × 10^−7^)*x* + (9.91 × 10^−9^), *R*^2^ = 0.19, *P* < 0.0001, for wild-type (WT); *y* = (− 9.21 × 10^−7^)*x* + (3.54 × 10^−8^), *R*^2^ = 0.09, *P* = 0.06, for Δ*mutS*; *y* = (− 1.67 × 10^−6^)*x* + (3.40 × 10^−8^), *R*^2^ = 0.20, *P* < 0.0001, for Δ*mutX*; *y* = (− 1.03 × 10^−6^)*x* + (4.33 × 10^−8^), *R*^2^ = 0.06, *P* = 0.04, for Δ*mutY*. **B** Repair efficiencies of the three pathways treated with variable penicillin concentration, error bars denote SE of a ratio, which was calculated by equation A1.19b on page 818 (Lynch and Walsh 1998), assuming no covariance between mutation rates of the wild-type and the knockout strains

In the wild-type MA lines, linear regression analysis revealed a weak negative correlation between the BPS mutation rates and the penicillin concentration (*R*^2^ = 0.19, *P* < 0.0001; Figs. 3A, B, 4A; Supplementary Fig. S1A), so did the Δ*mutX* (*R*^2^ = 0.20, *P* < 0.0001; Fig. 4A; Supplementary Figs. S1C, S2A, B) and the Δ*mutY* MA lines (*R*^2^ = 0.06, *P* = 0.04; Fig. 4A; Supplementary Figs. S1D, S2C, D). By contrast, there was no significant linear relationship between mutation rates and penicillin doses in the Δ*mutS* MA lines [(*R*^2^ = 0.09, *P* = 0.06; Figs. 3C, D, 4A; Supplementary Fig. S1B]. In addition, in all of the four strains, penicillin treatment did not change the rate of small-indels, the genome-wide distribution, or the mutation spectrum (Fig. 3B, D; Supplementary Figs. S2B, D, S3, S4).

In addition to analyzing BPSs and indels, we calculated the rates of structure variations (SVs). We detected in total 16 de novo SVs—all large-scale deletions—in all of the wild-type MA lines: 4, 4, 2, 2, 4 SVs for the groups treated with 0–0.008 ng/μL penicillin, using Breseq for SV-analysis and IGV for visual verification (Supplementary Tables S18, S19). No statistically significant linear relationship occurred between the SV rate and penicillin concentration (*R*^2^ = 0.06, *P* = 0.7). This pattern also extended to the other three strains (Δ*mutS*: *R*^2^ = 0.68, *P* = 0.08; Δ*mutX*: *R*^2^ = 0.0002, *P* = 0.98; Δ*mutY*: *R*^2^ = 0.17, *P* = 0.48; Supplementary Table S18).

We further explored whether penicillin treatment affected the efficiency of DNA repair systems. We found that there was no significant correlation observed between repair efficiency and penicillin doses (MMR repair efficiency: *R*^2^ = 0.56, *P* = 0.14; oxidative damage repair efficiency: Δ*mutX*, *R*^2^ = 0.26, *P* = 0.38; Δ*mutY*, *R*^2^ = 0.63, *P* = 0.11; Fig. 4B; Supplementary Table S16).

### Context-dependence of the wild-type mutation rate

As penicillin treatment did not alter BPS mutation spectra (Supplementary Fig. S4), we combined all 1510 BPSs from the control and penicillin-treated wild-type MA lines. This pooling strategy was used to enhance the statistical power of the analysis on the context-dependence of the mutation rate. The substantial number of mutations observed in the wild-type and those in the Δ*mutS* MA lines thus provided an opportunity to investigate the context-dependence of the mutation rate before and after the MMR repair.

**Fig. 5:**
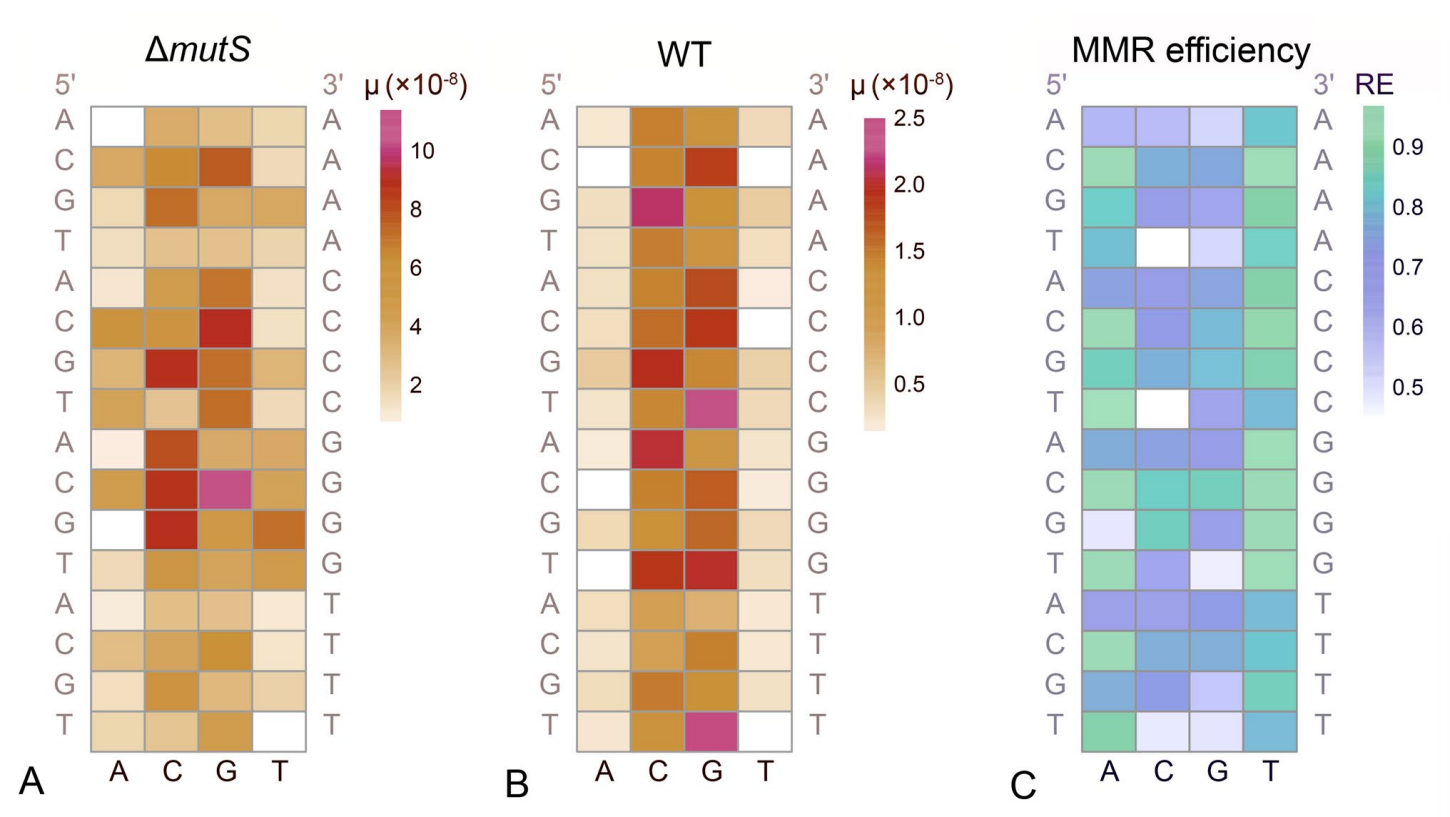
Context-dependence of the wild-type and the Δ*mutS* MA lines. The heatmaps show the mutation rate of each nucleotide context (the bases on the bottom are focal bases, the 5′ flanking nucleotides and the 3′ flanking nucleotides are on the left and right respectively). **A** The context-dependent mutation rate of the Δ*mutS* MA lines. **B** The context-dependent mutation rate of the wild-type MA lines. **C** The context-dependent MMR repair-efficiency; RE on the scale is the repair efficiency

In the MMR-deficient (Δ*mutS*) lines, the context-dependent mutation rates of nucleotides flanked by G/C indicated higher mutation rates compared to contexts lacking G/C flanking (Fig. 5A; Supplementary Table S20). Specifically, among all the 64 tri-nucleotide contexts (ranked from high to low), the top 10 contexts with the highest mutation rates were G/C-flanked. While in the wild-type lines, nine out of the top 10 contexts were G/C-flanked, except for 5′-T[G→N]T-3′, which ranked as the second highest. Additionally, the specific order of each context differed from that of the Δ*mutS* MA lines (where N represents any nucleotide) (Fig. 5B). These findings demonstrated that the context-dependence of mutation rates for focal nucleotides was maintained even after pre-mutations were repaired by MMR. However, the particular pattern was altered due to the differential context effects of MMR (with 79.2% repairing efficiency for contexts containing at least one G/C vs. 66.7% without any G/C; one-sided *t* test, *P* = 0.0026). Although flanking G/C nucleotides could elevate both the mutation rate and the repair efficiency, there was no association between the context-dependent mutation rate and repair efficiency of a focal nucleotide before MMR (i.e., in the Δ*mutS* lines) (*R*^*2*^ = − 0.0033, *P* = 0.38). Also, MMR preferentially repaired A/T focal nucleotides (with an 84.5% repair efficiency) compared to G/C ones (67.6%) (Fig. 5C; one-sided *t* test, *P* = 6.01 × 10^−7^), thus contributing to the genome-wide A/T mutation bias observed in the wild-type lines.

## Discussion

In this study, we explored the genomic mutational features of the Gram-positive pathogen *S. pneumoniae* D39. Our MA-WGS analysis revealed that it has the highest mutation rates of BPSs and indels among all studied bacteria with functional DNA repair systems. When cultured on non-stressing rich media and assessed using MA procedures, the mutation rate of *S. pneumoniae* D39 wild-type MA lines was estimated to be 0.02 per genome per cell division. This rate is orders of magnitude higher than those observed in all other non-mutator bacteria. Among previous studies on bacterial with functional MMR, spontaneous mutation rates have been estimated to range from 0.0003 to 0.0045 per genome per cell division. The highest rate was observed in the cold-adapted marine bacterium *Colwellia psychrerythraea*, while the lowest was documented in the insect-killing *Photorhabdus luminescens* (Supplementary Table S14) (Dettman et al. 2016; Dillon et al. 2015, 2017; Lee et al. 2012; Long et al. 2015, 2016, 2018a, b; Pan et al. 2021, 2022; Sung et al. 2012, 2015).

Based on the single nucleotide polymorphism sites (SNPs) of 195 natural strains (four–fold degenerate sites: 227,481; SNPs at these sites: 37,047), we calculated *π*_s_ (nucleotide diversity at silent sites or four-fold degenerate sites) of *S. pneumoniae* to be 0.023. The high *π*_s_ is supported, at least partially, by the multiple subpopulations existing in different parts of the host nasal cavity, such as the outer surface and the nasal tissues (Briles et al. 2005; Green et al. 2021). For haploid organisms, in mutation-drift equilibrium at neutrally evolving sites, the effective population size (*N*_e_) ≈ *π*_s_/2*μ* (Lynch et al. 2016; Sung et al. 2012). According to the above formula, we calculated *N*_e_ of *S. pneumoniae* to be 1.17 × 10^6^, one to two orders of magnitude lower than the *N*_e_ of other bacteria studied (Pan et al. 2022; Sung et al. 2012). The remarkably high mutation rate and the low *N*_e_ align well with the predictions of the drift-barrier hypothesis (DBH), as that a small *N*_e_ can be conductive to the evolution of high mutation rate according to DBH (Lynch 2010; Lynch et al. 2016; Sung et al. 2012). Besides the above, the natural competence of *S. pneumoniae* in transformation could contribute to the high mutation rate, potentially through frequent recombination events during colonization and infection within the host.

Our MA-WGS analysis revealed a distinct mutation spectrum for *S. pneumoniae* D39, deviating from those observed in most other non-pathogenic prokaryotes. The dominant types of mutations identified were G:C→A:T transitions and G:C→T:A transversions (Fig. 2B; Supplementary Fig. S4) (Dettman et al. 2016; Dillon et al. 2015; Kucukyildirim et al. 2016; Long et al. 2015, 2018a; Sung et al. 2015). G:C→T:A transversions can be predominantly caused by the oxidation of guanines in the DNA template strand, resulting from 8-oxo-G:A mismatches (Michaels et al. 1992). This pattern aligned with the fact that *S. pneumoniae* secretes a large amount of reactive oxygen species (ROSs) during vegetative growth and is catalase-deficient (Pericone et al. 2002). These distinctive mutational features highlight the significant influence of the idiosyncratic biology of *S. pneumoniae* on the spectrum of spontaneous mutations. Based on this observation, we hypothesize that guanines in the DNA template strand are more susceptible to oxidation compared to those in the nucleotide substrate pool. However, further exploration is required to support this hypothesis.

Multiple studies conducted in model organisms have established that mutation rates of individual nucleotides in DNA strands are influenced by their surrounding nucleotide context. Specifically, a higher G/C composition correlates with an increased mutation rate (Blake et al. 1992; Hess et al. 1994; Lee et al. 2012; Morton 2003; Pan et al. 2022). However, previous explorations have primarily focused on BPSs of mutator strains deficient in MMR, which have mutation rates orders of magnitude higher than those of the wild-type strains. This is because the number of de novo BPSs in wild-type MA lines was low and lacked the statistical power required for analyzing context-dependent mutation rates. The repair efficiency of MMR is also known to be context dependent, with a higher efficiency at nucleotides flanked by a higher G/C composition (Jones et al. 1987; Long et al. 2018a). As a result, it remains unclear whether the context-dependence of mutation rates persists after pre-mutations are repaired by the context-dependent MMR. In this study, we uncovered that among all the 64 tri-nucleotide contexts of the wild-type MA lines (ranked from high to low), nine out of the top 10 contexts were G/C-flanked, except for 5′-T[G→N]T-3′, which ranks as the second highest. These findings demonstrated that the context-dependence of mutation rates for focal nucleotides was maintained even after pre-mutations are repaired by MMR (Fig. 5A, B; Supplementary Table S20).

Compared with the BPS mutation rate of the wild-type *S. pneumoniae*, there was only ~ 3 times elevation in the BPS mutation rate of the Δ*mutS*, viz. MMR repairs 69.17% of pre-mutations at whole genome level. The genome-wide repair efficiency of MMR in *S. pneumoniae* was close to that observed in the Gram-positive bacterium *Deinococcus radiodurans*. In *D. radiodurans*, MMR showed an overall repair efficiency of 72.94%, with 75.33% of G:C→A:T and 87.79% of A:T→G:C transitions repaired at the whole genome level (Table 2; Fig. 1B; Supplementary Tables S2, S13, S16; Long et al. 2018a). The repair efficiency of MMR in *S. pneumoniae* was the lowest compared to all other bacteria studied with MA experiments. This provided a strong biological basis for the exceptionally high mutation rate in the wild-type strain. The function of MMR in most bacteria has undergone extensive refinement through natural selection, as MMR-deficient strains are known to be transient (Longerich et al. 1995). While natural selection primarily operates at the whole-bacterium level, it is possible that the cumulative fitness effects of mutations resulting from low MMR efficiency could be counteracted by multiple layers of other DNA repair mechanisms (Lynch 2012). For example, these may include highly efficient oxidative damage repair systems, akin to those observed in *D. radiodurans* (Krisko and Radman 2013; Long et al. 2015).

Given the natural absence of catalase in *S. pneumoniae*, it is reasonable to assert that pathways linked to MutX and MutY, responsible for fixing ROS-caused oxidative damages, play crucial roles in repairing mutations originating from oxidized guanines present in the cellular nucleotide pool or the DNA strands (Mejean et al. 1994; Michaels et al. 1992; Tajiri et al. 1995). Indeed, repair efficiencies were high in specific mutation categories. MutX demonstrated a robust repair efficiency of 96.7% for A:T→C:G transversions, mutations typically caused by oxidized guanines in the cellular nucleotide pool. Similarly, MutY had a high repair efficiency of 90.52% for G:C→T:A transversions, mutations typically arising from oxidized guanines in the DNA strands. It has been reported that when infecting hosts, *S. pneumoniae* can utilize catalase from co-existing pathogens to reduce its cellular ROS levels, and secrete ROS to inhibit the surrounding pathogens, providing a competitive advantage for its growth (Bogaert et al. 2004; Dahiya and Speck 1968; Pericone et al. 2000). The high levels of secreted ROSs can also assist in host invasion by destroying specific barriers or tissue cells (Weiser et al. 2018; Yesilkaya et al. 2013), suggesting that the absence of catalase in this pathogen is a double-edged sword rather than being entirely harmful. Therefore, in addition to mutation pressure arising from mismatches during DNA replication, it is plausible that *S. pneumoniae* may experience additional mutation pressure due to oxidative DNA damages.

Penicillin has been used to fight *S. pneumoniae* infections for ~ 80 years, but penicillin-resistant strains have now spread globally (Mandell et al. 2007; Watson et al. 1993; Yu et al. 2003). Resistance can arise through spontaneous mutations, including point mutations and horizontally transferred genes, or induced mutations caused by physical or chemical mutagens (Munita and Arias 2016; Zhang et al. 2023). In this study, we found that penicillin treatment did not elevate the mutation rates of *S. pneumoniae* at any mutation scale. Our previous research demonstrated that treatment with the fluoroquinolone antibiotic norfloxacin can increase genomic mutation rates in *E. coli* by inducing the SOS stress response. This SOS response is triggered by DNA breaks resulting from norfloxacin inhibiting DNA gyrase, leading to the introduction of more mutations by the low-fidelity DNA polymerases in treated cells compared to untreated ones (Long et al. 2016; Napolitano et al. 2000). As that penicillin does not cause DNA damages directly and *S. pneumoniae* naturally lacks the SOS response pathway, it supports the stress-induced mutagenesis hypothesis from a new perspective, suggesting that the mutation rate is not elevated by antibiotic stress in the absence of the SOS response pathway (Gasc et al. 1980; Napolitano et al. 2000; Sauvage et al. 2008; Waxman and Strominger 1983). Stress-induced mutagenesis may, therefore, be limited to agents specifically activating the SOS response pathway by inducing DNA damage, rather than being extended to general stress conditions.

In comparison to other model bacteria, such as *E. coli*, *S. pneumoniae* demonstrates a greater specialization in habitats and presents higher costs and labor/lab requirement for conducting long-term and large-scale evolution experiments due to its classification as a biosafety level 2 pathogen. Nevertheless, this study paves the way for further exploration of the evolutionary trajectory of *S. pneumoniae*, which is listed as one of the 12 priority pathogens by the World Health Organization (Asokan et al. 2019). This bacterium is responsible for multiple diseases and demonstrates a propensity for developing multidrug resistance (Weiser et al. 2018). The two orders of magnitude higher genomic mutation rate observed in this MMR-functional pathogen, compared to the mutation rates of most reported bacteria, presents a valuable opportunity for testing hypotheses regarding mutation rate evolution.

### Supplementary Information

Below is the link to the electronic supplementary material.

Supplementary file1 (XLSX 1458 KB)

Supplementary file2 (DOCX 1301 KB)

## Data Availability

All sequencing data in this research are available at NCBI SRA with the BioProject number of PRJNA781759.
