# Correction: Spontaneous mutations and mutational responses to penicillin treatment in the bacterial pathogen *Streptococcus pneumoniae* D39

**DOI:** 10.1007/s42995-024-00232-2

**Published:** 2024-05-22

**Authors:** Wanyue Jiang, Tongtong Lin, Jiao Pan, Caitlyn E. Rivera, Clayton Tincher, Yaohai Wang, Yu Zhang, Xiang Gao, Yan Wang, Ho-Ching T. Tsui, Malcolm E. Winkler, Michael Lynch, Hongan Long

**Affiliations:** 1 Institute of Evolution and Marine Biodiversity, KLMME, Ocean University of China, Qingdao, 266003 China; 2 Laboratory for Marine Biology and Biotechnology, Laoshan Laboratory, Qingdao, 266237 China; 3 Department of Biology, Indiana University, Bloomington, IN 47405 USA; 4 School of Mathematics Science, Ocean University of China, Qingdao, 266000 China; 5 State Key Laboratory of Microbial Technology, Microbial Technology Institute, School of Life Science, Shandong University, Qingdao, 266237 China; 6 Biodesign Center for Mechanisms of Evolution, Arizona State University, Tempe, AZ 85281 USA

**Correction: Marine Life Science and Technology** 10.1007/s42995-024-00220-6

Due to technical errors, Ho-Ching T. Tsui and Malcolm E. Winkler, both collaborators who made substantial contributions to this work and were previously acknowledged, did not receive emails regarding authorship consent and were inadvertently omitted from the author list. We have now rectified this oversight and added them to the author list.

We have also made the following updates:

- Rewrote the first paragraph of the Materials and methods section.
- In the current third paragraph of Materials and methods, we replaced “Cells were cultured for 14 h in BHI broth and then serially diluted to a suitable density” with “cells were inoculated from frozen glycerol stocks into BHI broth, serially diluted, and incubated 12–15 h statically at 37 °C in an atmosphere of 5% CO_2_. The next day, cultures at OD620 ≈ 0.1–0.4 were diluted to OD620 ≈ 0.005 in BHI broth.”
- Supplementary Information has been updated:
- Strain, primer information and notes on strain gene deletion procedures were also added to Supplementary Table S1.
- Six new references (Lanie et al. J Bacteriol 2007, Ramos-Montanez Mol Microbiol 2008, Slager et al. Nucleic Acids Res 2018, Tsui et al. mBio 2011, Tsui et al. Mol Microbiol 2016, Tsui et al. Mol Microbiol 2014) were cited and added to the References section.
- The Acknowledgement section was correspondingly updated by removing the sentence “We truly appreciate the kind help of Prof. Tiffany Tsui and Prof. Malcolm Winkler (Indiana University at Bloomington) in generously providing the *S. pneumoniae* D39 strains and lab space for culturing and transferring the cell lines.” Additionally, “National Institutes of Health award (RG-GM122566)” was replaced with “National Institutes of Health award (RG-GM122566 to ML) and (R35-GM131767 to MEW)”.
- The Contribution section was updated by adding the contributions of new authors Ho-Ching T. Tsui and Malcolm E. Winkler.

### Supplementary Information

Below is the link to the electronic supplementary material.

Supplementary file1 (XLSX 1458 KB)

Supplementary file2 (DOCX 1301 KB)

