# Supplementary material for: Spontaneous mutations and mutational responses to penicillin treatment in the bacterial pathogen Streptococcus pneumoniae D39
Source: Mar Life Sci Technol. 2024 Apr 16;6(2):198–211. doi: 10.1007/s42995-024-00220-6 (PMC11136922; doi:10.1007/s42995-024-00220-6)
Supplement: Supplementary file 2 — Supplementary file2 (DOCX 1301 KB) [file 42995_2024_220_MOESM2_ESM.docx]

**Supplementary Information**

**Spontaneous mutations and mutational responses to penicillin treatment in the bacterial pathogen *Streptococcus pneumoniae* D39**

Wanyue Jiang^1,2,#^, Tongtong Lin^1,#^, Jiao Pan^1,#^, Caitlyn E. Rivera^3^, Clayton Tincher^3^, Yaohai Wang^1^, Yu Zhang^4^, Xiang Gao^5^, Yan Wang^1^, Ho-Ching T. Tsui^3^, Malcolm E. Winkler^3^, Michael Lynch^6^, Hongan Long^1,2,*^

1. Institute of Evolution and Marine Biodiversity, KLMME, Ocean University of China, Qingdao 266003, China

2. Laboratory for Marine Biology and Biotechnology, Laoshan Laboratory, Qingdao 266237, China

3. Department of Biology, Indiana University, Bloomington, Indiana 47405, USA

4. School of Mathematics Science, Ocean University of China, Qingdao 266000, China

5. State Key Laboratory of Microbial Technology, Microbial Technology Institute, School of Life Science, Shandong University, Qingdao 266237, China

6. Biodesign Center for Mechanisms of Evolution, Arizona State University, Tempe, Arizona 85281, USA

# These authors contributed equally to this work.

* Corresponding author, Email: longhongan@ouc.edu.cn

**Supplementary Figures**

**
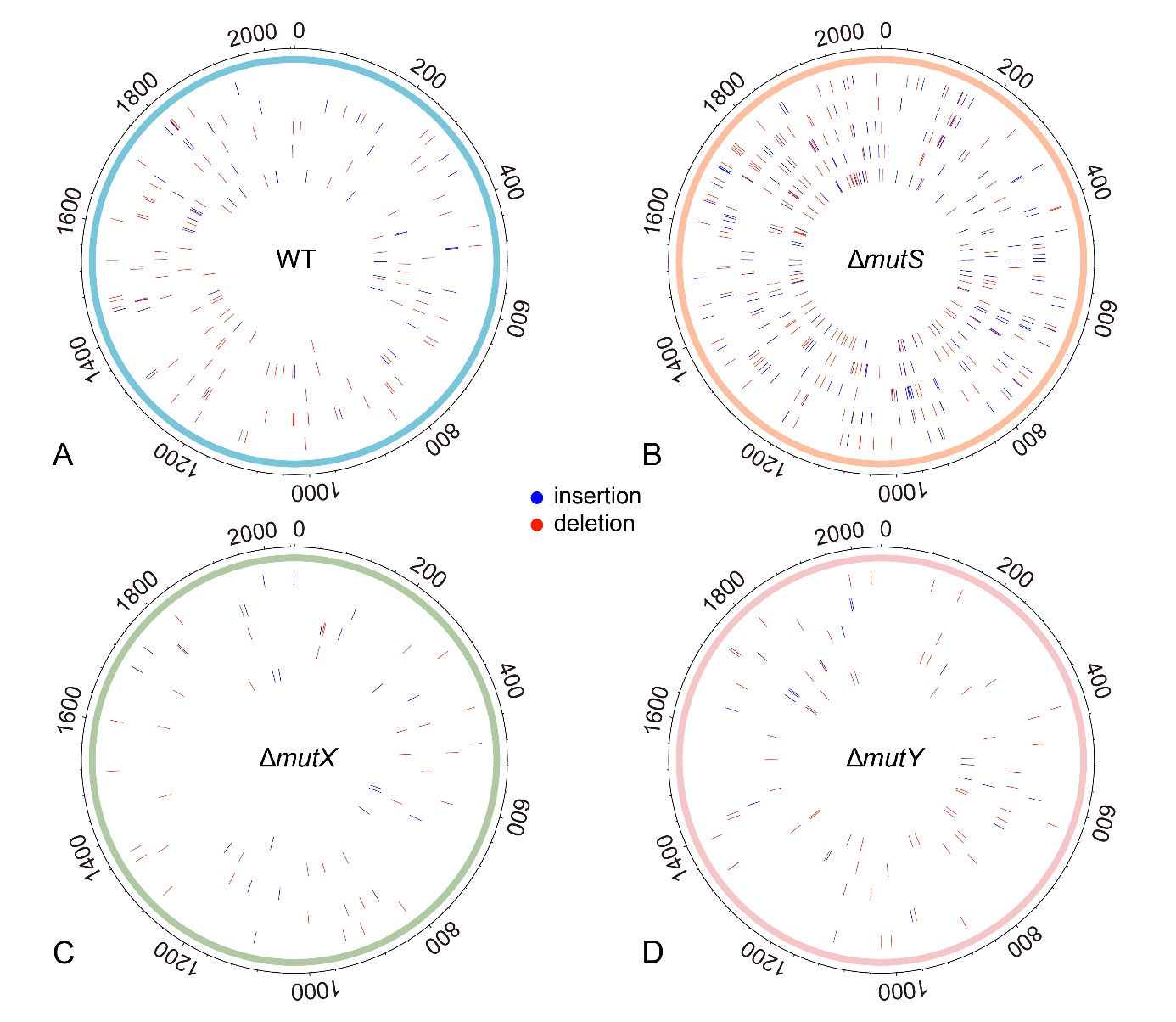
**

**Supplementary Fig. S1 The indels (insertions and deletions) distribution of MA lines with or without penicillin treatment (A**–**D).** Genome coordinates are in kbp. Circles with colored tiles from the outmost on represent indels of 0, 0.002, 0.004, 0.006 and 0.008ng/μL-penicillin-treated MA lines.


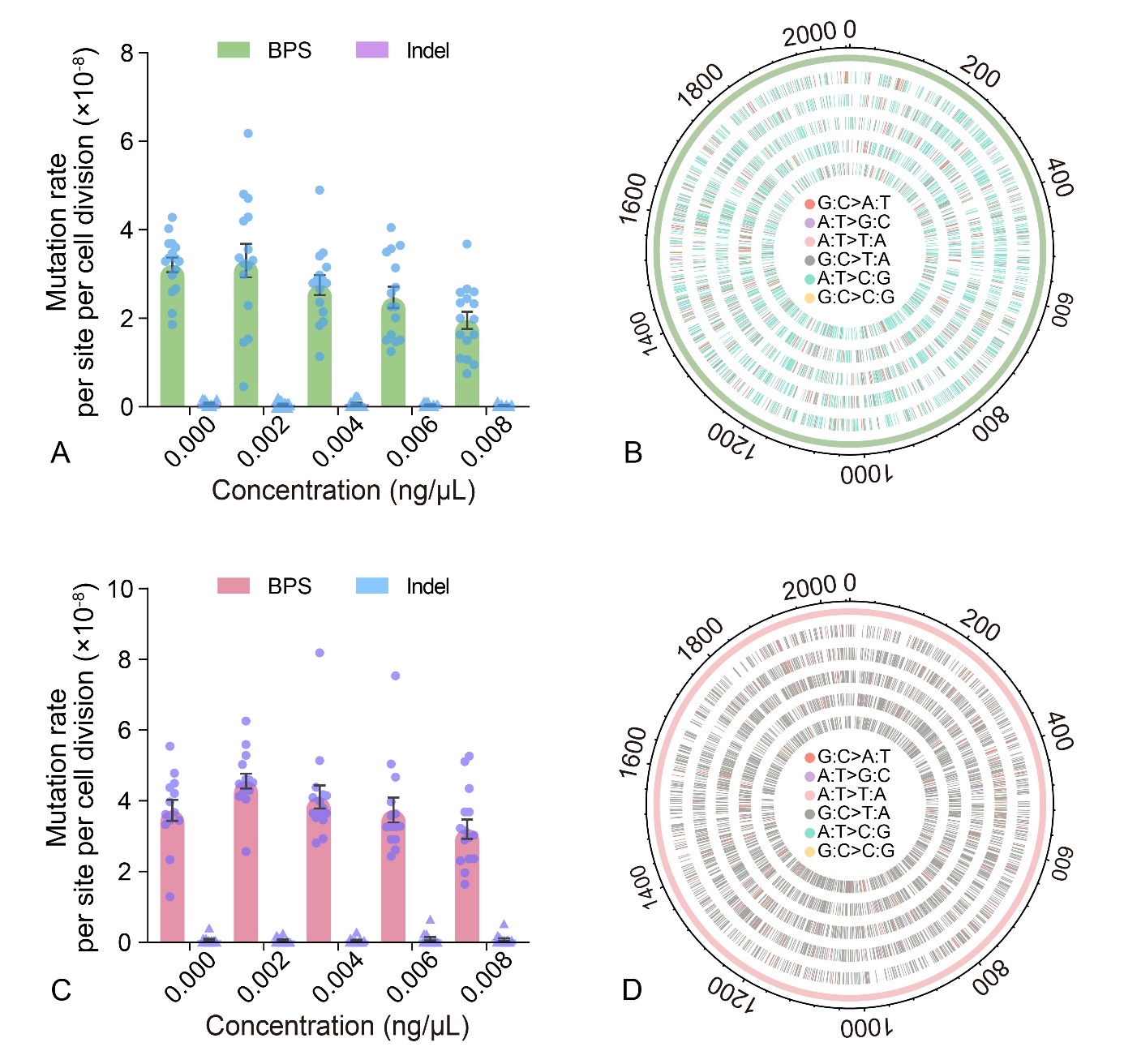


**Supplementary Fig. S2 Mutational features of the Δ*mutX* and the Δ*mutY* MA lines.** **A** BPS (base-pair-substitutions) and indel (insertions and deletions) mutation rates treated with different penicillin concentrations of the Δ*mutX* MA lines**. B** The BPSs distribution on the whole genome of the Δ*mutX* MA lines. **C** BPS and indel mutation rates treated with different penicillin concentrations of the Δ*mutY* MA lines. **D** The BPSs distribution on the whole genome of the Δ*mutY* MA lines. The dots and the triangles represent BPS and indel mutation rate of each MA line respectively; error bars denote SE (**A, C**). Circles with colored tiles from the outmost on represent BPSs of 0, 0.002, 0.004, 0.006 and 0.008ng/μL-penicillin-treated MA lines (**B, D**).


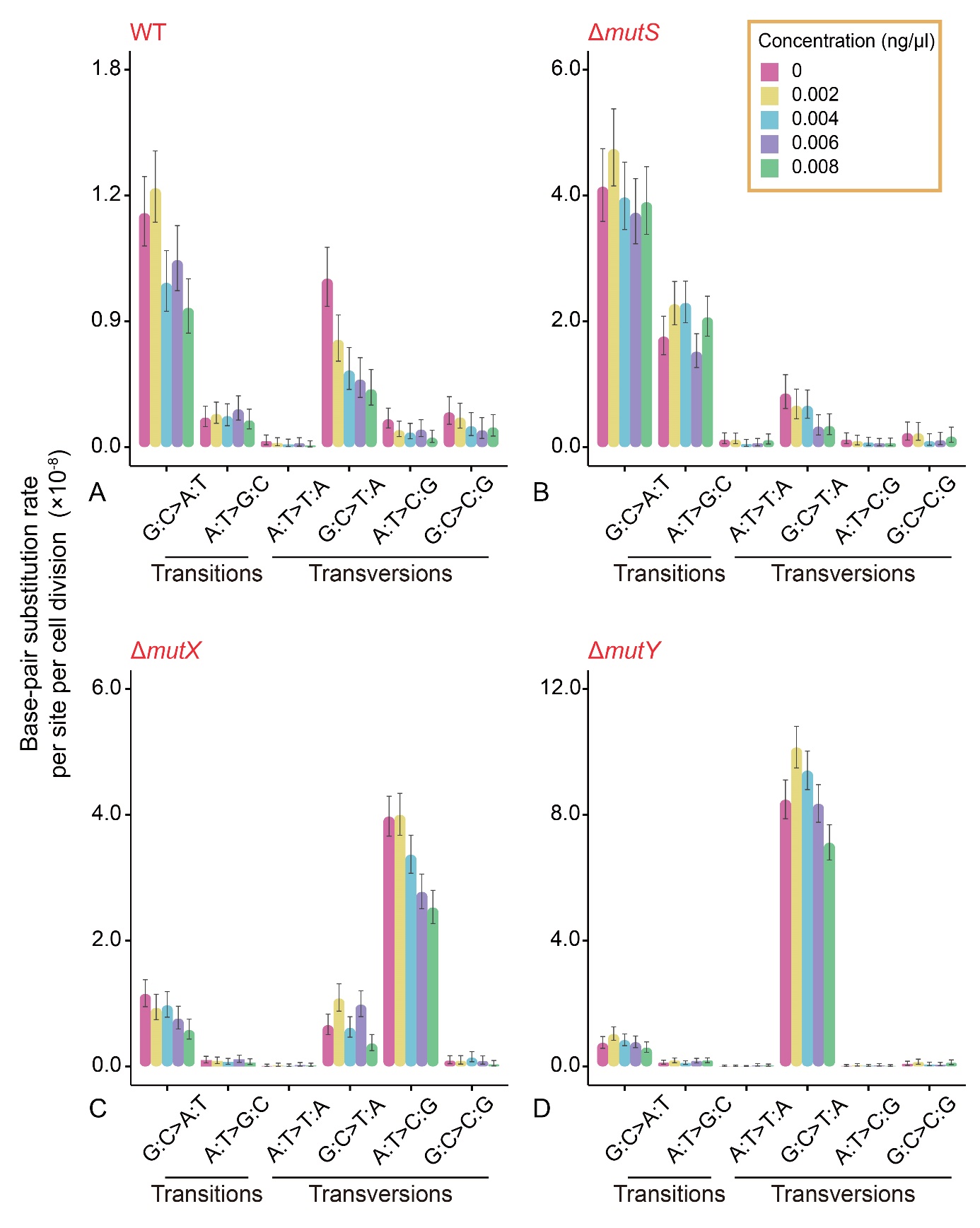


**Supplementary Fig. S3 Mutation spectra of the MA lines treated with different penicillin concentrations. A** WT; **B** Δ*mutS*; **C** Δ*mutX*; **D** Δ*mutY*. Error bars denote 95% Poisson confidence intervals.


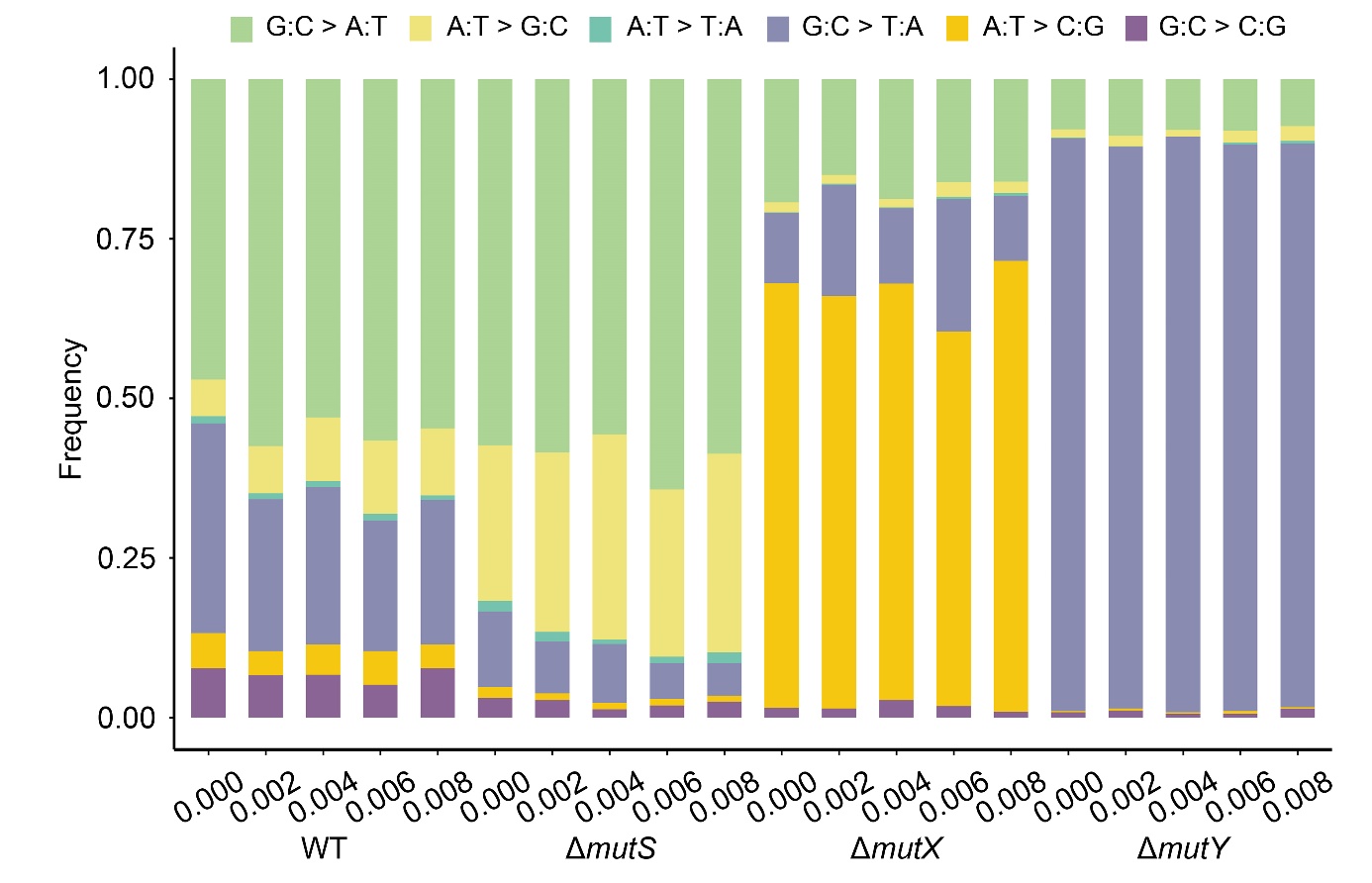


**Supplementary Fig. S4** **Frequencies of the six mutation types in the WT and the three mutants MA lines of the five groups** (0, 0.002, 0.004, 0.006 and 0.008ng/μL-penicillin-treated groups)**.**
